# Mitral valve surgery for atrial functional mitral regurgitation: predicting recurrent mitral regurgitation and mid-term outcome

**DOI:** 10.1007/s11748-022-01793-8

**Published:** 2022-03-03

**Authors:** Naonori Kawamoto, Satsuki Fukushima, Satoshi Kainuma, Ayumi Ikuta, Naoki Tadokoro, Takashi Kakuta, Tomoyuki Fujita

**Affiliations:** Department of Cardiovascular Surgery, National Cerebral and Cardiovascular Center, 6-1 Kishibe-Shinmachi, Suita, Osaka 564-8565 Japan

**Keywords:** Atrial fibrillation, Atrial functional mitral regurgitation, Mitral valve repair, Mitral valve replacement, Long-term outcome

## Abstract

**Objectives:**

This study aimed to elucidate the mid-term outcomes and risk factors for recurrent mitral regurgitation after mitral valve (MV) surgery for atrial functional mitral regurgitation (AFMR).

**Methods and results:**

We retrospectively analyzed data of 50 consecutive patients (median age 74 years; 29 men) who underwent mitral valve surgery for AFMR between January 2001 and January 2019. Mean atrial fibrillation duration was 12 years. During the follow-up period of 4.6 ± 4.4 years, 5 cardiac-related deaths were identified. Five- and 10-year freedom from cardiac-related death rate for all patients was 88.4% and 78.6%. In total, 42 patients underwent MV repair with mitral annuloplasty and 8 underwent MV replacement. Five- and 10-year freedom from cardiac-related death rate in patients who underwent MV repair was 93.1% and 82.7%, which was better than MV replacement (log rank *p* = 0.04). During the follow-up period, MR recurrence rate was 16.8% at 5 and 10 years for the patients who underwent MV repair. Univariate analysis showed that partial band annuloplasty and preoperative elevated left ventricular end-systolic volume index were risk factors for recurrent MR after MV repair. Multivariate analysis identified partial band annuloplasty as the independent predictor for recurrent MR during long-term follow-up after MV repair for AFMR.

**Conclusion:**

Patients who underwent MV repair for AFMR could have an acceptable mid-term outcome. However, MVR might not improve the mid-term outcome in patients with AFMR. The use of partial bands for mitral annuloplasty would not be recommended in terms of recurrent MR mid-term.

**Supplementary Information:**

The online version contains supplementary material available at 10.1007/s11748-022-01793-8.

## Introduction

Atrial functional mitral regurgitation (AFMR) is known to have been caused by an enlarged left atrium and insufficient leaflet remodeling in patients with atrial fibrillation (AF) despite having preserved left ventricular systolic function [1, 2].

The latest update of Japanese guideline on the management of valvular heart disease clearly distinguished between secondary MR and AFMR [3]. Mitral valve surgery is recommended for patients having AFMR with frequent heart failure despite of medical therapy. The 2021 ESC/EACTS guideline [4] also mentioned the patients with AFMR might be more effectively treated by ring annuloplasty, but evidence is still limited. For AFMR, studies have shown good short- and mid-term outcomes after MV repair, with and without complex repair, including secondary chordal cutting, posterior leaflet extension, and posterior left atrial (LA) wall plication [5–9]. However, no study has previously investigated the clinical outcomes of MV replacement for AFMR. Furthermore, few studies have demonstrated the mid-term outcomes and recurrent MR after surgical correction for AFMR [10]. Therefore, this study aimed to elucidate the mid-term outcomes after surgical correction including MV repair and MV replacement for AFMR. We also investigated the risk factor for recurrent MR after mitral valve repair for AFMR.

## Materials and methods

### Patients

**Fig. 1:**
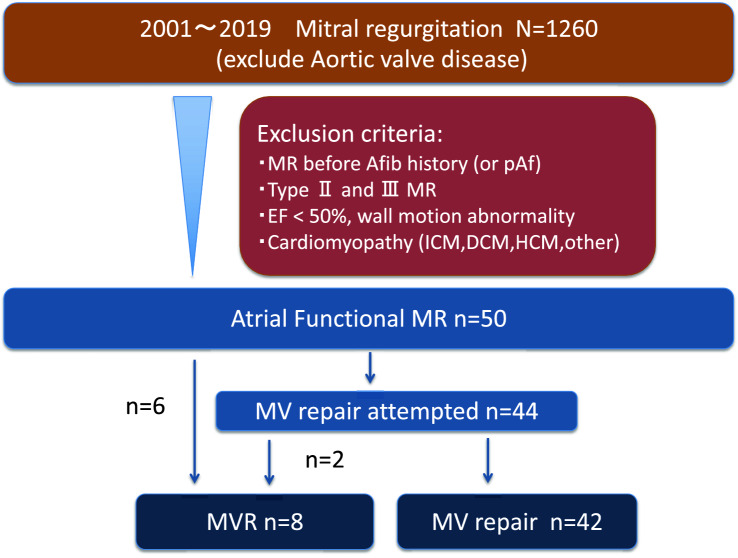
Study group identification. Patients with aortic valve disease were excluded. Clinically, the patients were first identified with atrial fibrillation (confirmed by electrocardiogram) before MR appeared. Patients were excluded if the cause of the MR was rheumatic heart disease, degenerative mitral valve disease, mitral valve leaflet perforation, ischemic heart disease or cardiomyopathy. Patients with an ejection fraction < 50% or a ventricular wall motion abnormality were also excluded. *MR* mitral regurgitation

In this study, we included patients who underwent MV surgery for AFMR between January 2001 and January 2019. We retrospectively analyzed the data of a prospectively enrolled cohort, which included 50 consecutive patients with AFMR (Fig. 1). Patients with aortic valve disease were excluded.

Clinically, the patients had first been identified with AF (confirmed by electrocardiogram) before the MR appeared. Patients were excluded if the cause of the MR was rheumatic heart disease, degenerative MV disease, MV leaflet perforation, ischemic heart disease, or cardiomyopathy. Patients with an ejection fraction of < 50% or a ventricular wall motion abnormality were also excluded.

The study protocol conforms to the ethical guidelines of the Declaration of Helsinki as reflected in a priori approval by the institution’s human research committee.

Median age of patients was 74 years (range 65.7–77 years), and 29 (58%) patients were men. Among the 50 patients, 42 (82%) had paroxysmal AF, and the mean duration of the AF was 12 years (range, 5–21 years) (Table 1).

**Table 1:** Patient backgrounds and preoperative examinations

	All	MV repair	MVR	*P* value
Number of patients	50	42	8	
Male	29 (58%)	26 (62%)	3 (37%)	0.19
BSA (m^2^)	1.57 ± 0.16	1.59 ± 0.14	1.51 ± 0.24	0.2
Age	74 (65.7–77)	73.5 (64.5–77.3)	74.5 (69.3–77)	0.5
HT	33 (66%)	26 (62%)	7 (87%)	0.16
HL	12 (24%)	10 (23%)	2 (25%)	0.94
DM	2 (4%)	2 (4.7%)	0	0.52
FEV1% < 70% (%)	21 (40%)	18 (43%)	3 (37%)	0.77
%VC < 80% (%)	7 (14%)	6 (14%)	1 (12%)	0.89
Thyroid disease	7 (14%)	5 (12%)	2 (25%)	0.32
Creatinine level	0.88 (0.76–1.17)	0.88 (0.76–1.1)	0.89 (0.77–1.4)	0.71
CKD	9 (18%)	7 (17%)	2 (25%)	0.57
Persistent Af	41 (82%)	35 (83%)	7 (75%)	0.57
Afib duration (year)	12 (5–21)	13 (6–21)	8.5 (2.25–14.7)	0.19
Angiographical findings				
Mean PA pressure (mmHg)	21 (18–25.5)	21 (18–25)	20 (15–27)	0.76
PCWP (mmHg)	13 (10–17.8)	13 (10–17)	16 (6–20)	0.84
Mean RA pressure (mmHg)	6 (4–8)	7.1 ± 0.8	6.2 ± 2.8	0.3
CI (L/min/m^2^)	2.7 ± 0.7	2.7 ± 0.1	3.0 ± 0.2	0.17
Echocardiographical findings				
MR grade 4	16 (32%)	11 (26%)	5 (62.5%)	0.04
LVDd (mm)	57 ± 8.5	57 ± 8.3	61 ± 9.4	0.25
LVEDV (ml)	160 (123–195)	156 (115–193)	176 (136–221)	0.24
LVEDVI (ml/m^2^)	101 (76–124)	103 ± 35	126 ± 35	0.07
LVDs (mm)	38 ± 6.8	38 ± 6.3	42 ± 8.3	0.22
LVESV (ml)	61 (44–85)	60 (43–84)	70 (56–86)	0.22
LVESVI (ml/m^2^)	38 (30–51)	37 (26–49)	50 (35–63)	0.04
LA dimension (mm)	59 (53–72)	62 ± 14	61 ± 13	0.71
LAV (ml)	194 (135–410)	194 (129–407)	230 (157–425)	0.66
LAVI (ml/m^2^)	122 (84–258)	117 (78–258)	135 (102–260)	0.51
TRPG (mmHg)	28 (22–40)	28 (22–38)	32 (23–43)	0.7
EF (%)	69 ± 7.8	70 ± 7.8	67 ± 7.7	0.21
TR ≥ 3	17 (34%)	13 (31%)	4 (50%)	0.29
Mitral annular dilation	50 (100%)	42 (100%)	8 (100%)	1
Posterior leaflet tethering	11	7 (17%)	4 (50%)	0.037

*BSA* body surface area, *HT* hypertension, *HL* hyperlipidemia, *DM* diabetes mellitus, *FEV1%* forced expiratory volume in 1 s, *%VC* % vital capacity, *CKD* chronic kidney disease, *pAf* paroxysmal atrial fibrillation, *Afib* atrial fibrillation, *PA* pulmonary artery, *PCWP* pulmonary capillary wedge pressure, *RA* right atrial, *CI* cardiac index, *MR* mitral regurgitation, *LVDd* left ventricular diastolic diameter, *LVEDV* left ventricular end-diastolic volume, *LVEDVI* left ventricular end-diastolic volume index, *LVDs* left ventricular systolic diameter, *LVESV* left ventricular end-systolic volume, *LVESVI* left ventricular end-systolic volume index, *LA* left atrial, *LAV* left atrial volume, *LVVI* left atrial volume index, *TRPG* peak tricuspid regurgitant pressure gradient, *EF* ejection fraction, *TR* tricuspid regurgitation

### Surgical procedures

The surgical indications for AFMR included a preoperative grade 3 MR with a previous history of hospitalization for heart failure caused by AFMR, or grade 4 MR with or without symptoms.

MV replacement was performed to avoid multiple corrective attempts and longer cardiopulmonary bypass times and in cases when difficult repair situations were expected, such as leaflet tethering and insufficient leaflet remodeling despite enlarged annulus. MV annuloplasty using a prosthetic full ring or partial band was performed on all patients undergoing MV repair. Operative data are shown in Table 2. Of the 44 candidates for MV repair, 42 had their MR successfully repaired and 2 required MV replacement after MV repair was attempted. Although elective MV replacement was scheduled for 6 patients, 8 patients consequently underwent MV replacement. The annuloplasty prosthesis size was selected based on the intertrigonal distance measured, without “downsizing,” with the sizer provided. When the intertrigonal distance was obscured, the length of the anterior leaflet was used to select the prosthesis size.

**Table 2:** Surgical outcome and postoperative course

	*N* = 50	MV repair *n* = 42	MVR *n* = 8	*P* value
Mitral annuloplasty	42	42 (100%)		
Ring size (mm)	28 (27–30)	28 (27–30)		
Partial band	5 (11.9%)	5 (11.9%)		
Flexible ring/band	10 (23%)	10 (23%)		
Chordal replacement	3 (7.2%)	3 (7.2%)		
Chordal cutting	1 (2.3%)	1 (2.3%)		
Cleft suture	5 (12%)	5 (12%)		
Edge to edge repair	1 (2.3%)	1 (2.3%)		
Mechanical valve	3 (37.5%)		3 (37.5%)	
Concomitant procedure				
With Maze	20 (40%)	18 (43%)	2 (25%)	0.34
With TAP	40 (80%)	34 (81%)	6 (75%)	0.69
With TVR	1 (2%)	1 (2.4%)	0	0.65
With LAA closure	33 (66%)	27 (64%)	6 (75%)	0.55
With PFO closure	2 (5%)	1 (2.4%)	1 (12.5%)	0.18
With PMI	5 (10%)	5 (12%)	0	0.12
Post-op course				
Length of hospital stay (day)	13 (11–17.5)	13 (11–17.5)	13 (11–21)	0.55
Hospital death	1 (2%)	0	1 (12.5%)	0.02
Respiratory failure	2 (4%)	1 (2.4%)	1 (2.3%)	0.24
Cerebrovascular event	1 (2%)	1 (2.4%)	0	0.67
Reexploration	2 (4%)	2 (4.8%)	0	0.55
Renal failure	1 (2%)	1 (2.4%)	0	0.67
Sinus rhythm	16 (32%)	14 (77%)	2 (100%)	0.45

*TAP* tricuspid annuloplasty, *TVR* tricuspid valve replacement, *LAA* left atrial appendage, *PMI* pacemaker implantation

Concomitant cardiac procedures included tricuspid annuloplasty in 40 patients (80%), tricuspid valve replacement in 1 (2%), maze procedure in 20 (40%), and LA appendage closure in 33 (66%).

### Echocardiography

Echocardiography was performed preoperatively, postoperatively, and during follow-up. Doppler echocardiography classified the MR grades as follows: 0, none; 1, trivial; 2, mild; 3, moderate; and 4, severe. Severe MR was defined when Doppler echocardiography detected a central jet with an MR of > 40% of the LA area or a holosystolic eccentric jet with MR, a vena contracta > 0.7 cm, a regurgitant volume > 60 mL, a regurgitant fraction > 50%, or an effective regurgitant orifice > 0.40 cm^2^. The categories of trivial, mild, and moderate MR were individually graded by an expert engineer and expert doctor.

We defined recurrent MR as moderate or severe MR after the initial MV operation.

### Statistical analysis

Categorical data in the Tables are presented as numbers (percentages). We checked the normality distributions for continuous data using the Shapiro–Wilk test, and we presented normally distributed continuous data as mean ± standard deviation. Alternatively, we presented the data as median and interquartile range. We compared the findings in the Tables through univariate analyses using the chi-squared test for categorical variables, Student’s *t* test for normally distributed continuous variables, and Mann–Whitney *U* test for non-normally distributed continuous variables. We used the cox hazard model to predict the risk factors for recurrent MR and heart failure after MV repair. A stepwise regression method was used to select significant variables from variables with a univariate *P* value of < 0.2. The Kaplan–Meier method was used to identify the freedom from cardiac-related death, heart failure, and recurrent MR rates. These rates were then compared between the groups using the log rank test. A *P* value of < 0.05 was considered statistically significant. All statistical analyses were performed using the JMP 14 statistical software package (SAS Institute, Inc., Cary, NC).

## Results

### Patient backgrounds and preoperative examinations

The follow-up rate was 96%, and the mean follow-up period was 4.6 ± 4.4 years. The patients’ baseline characteristics and preoperative examinations are described in Table 1. Median age of the patients was 74 years (range 65–77 years) and 29 (58%) were men. The number of patients with paroxysmal AF was 8 (18%), and the mean duration of the AF was 12 years (5–21 years) (Table 1).

There were no significant differences in the past medical history and angiographic findings between the patients in the MV repair and MV replacement groups. Angiography revealed that the mean pulmonary artery pressure for both groups was similarly elevated with no significant intergroup differences (*P* = 0.76). Echocardiography outcomes indicated that the number of patients with preoperative grade 3 MR was significantly higher in the MV repair group than in the MV replacement group (*P* = 0.04). Patients who underwent MV replacement had a significantly higher mean LVESVI than those who underwent MV repair (50 mL/m^2^ vs. 37 mL/m^2^, p = 0.04). There was a significant difference in the posterior leaflet tethering identified in 7 patients who underwent MV repair and in 4 patients who underwent MV replacement (*P* = 0.037).

### Surgical outcomes and postoperative course

The surgical findings for all patients are listed in Table 2. In MV replacement, the choice of prosthesis was determined according to patient age, special request, and presence of comorbidities, and 3 mechanical and 5 bioprosthetic valves were subsequently implanted. In MV repair, partial bands were implanted in 5 patients and full rings were implanted in 37. Flexible rings/bands were used for 10 patients, and the median ring size was 28 mm.

There was no significant difference in concomitant procedures.

Postoperative echocardiographic findings are presented in Table 3. Patients who underwent MV replacement had a significantly higher mean LVESVI than those who underwent MV repair (58 mL/m^2^ vs. 35 mL/m^2^, *P* = 0.037). The ejection fraction was 47% in patients undergoing MV replacement, which was lower than the 53% in those undergoing MV repair, but the difference was not significant (*P* = 0.19). The mean trans-mitral pressure gradient after MV replacement was relatively higher than that after MV repair, but the difference was not significant (*P* = 0.11). Other echocardiographic findings were similar between MV repair and MV replacement.

**Table 3:** Postoperative echocardiographic findings

	*n* = 50	MV repair *n* = 42	MVR *n* = 8	*P*
MR ≥ 2	2 (4%)	2 (4.8%)	0	0.55
LVDd	53 ± 7.2	52 ± 6.9	55 ± 9.1	0.7
LVEDV	135 (112–173)	135 (112–173)	129 (112–180)	0.7
LVEDVI	89 ± 24	86 ± 23	101 ± 27	0.16
LVDs	38 ± 7.2	37 ± 6.9	42.4 ± 7.9	0.11
LVESV	58 (44–78)	56 (41–76)	78 (50–118)	0.12
LVESVI	35 (28–51)	35 (28–44)	58 (34–74)	0.037
LA Dimension	53 ± 11	54 ± 10	53 ± 15	0.6
LAEDV	162 (103–254)	163 (106–255)	162 (83–257)	0.6
LAEDVI	95 (67–155)	94 (68–156)	110 (57–159)	0.59
TRPG	22.5 (19.7–28)	22.5 (19.3–28.8)	23.5 (20.5–27)	0.8
EF	52 ± 11	53 ± 11	47 ± 11	0.19
TR ≥ 2	9 (18%)	8 (19%)	1 (12.5%)	0.64
mPG	3.6 ± 1.3	3.4 ± 1.3	4.1 ± 1.1	0.11
mPG ≥ 5	9 (18%)	6 (14%)	3 (38%)	0.1

*MR* mitral regurgitation, *LVDd* left ventricular diastolic diameter, *LVEDV* left ventricular end-diastolic volume, *LVEDVI* left ventricular end-diastolic volume index, *LVDs* left ventricular systolic diameter, *LVESV* left ventricular end-systolic volume, *LVESVI* left ventricular end-systolic volume index, *LA* left atrial, *TRPG* peak tricuspid regurgitant pressure gradient, *EF* ejection fraction, *TR* tricuspid regurgitation, *mPG* mean trans-mitral pressure gradient

There was one case of hospital death among the patients who underwent MV replacement. Mean duration of hospital stay was similar (13 days) for the two groups. During hospitalization, 1 patient undergoing MV repair experienced a cerebrovascular event and respiratory failure.

### Recurrent MR, heart failure, cerebral complication and survival during follow-up

**Fig. 2:**
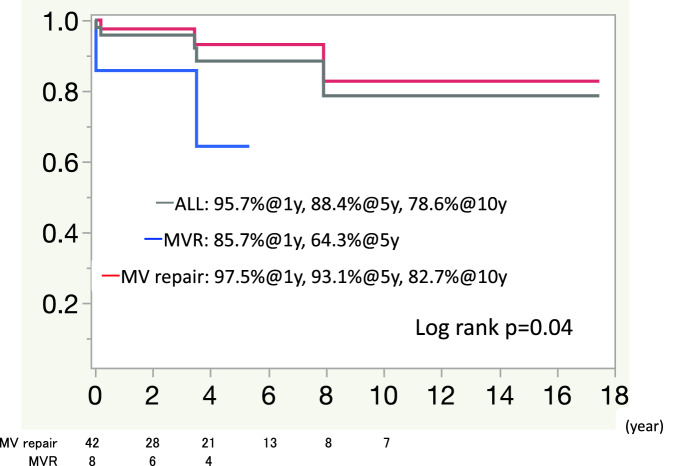
Freedom from cardiac death in the study group. The freedom from cardiac death rate for all patients was 95.7% at 1 year, 88% at 5 years, and 78.6% at 10 years. The 1- and 5-year freedom from cardiac-related death rates was 85.7% and 64.9%, respectively, in the MVR group. The 1-, 5-, and 10-year freedom from cardiac-related death rates was 97.5%, 93.1%, and 82.7%, respectively, in the MV repair group (log rank *p* = 0.04). *MVR* mitral valve replacement, *MV* *repair* mitral valve repair

The freedom from cardiac death rate for all patients was 95.7% at 1 year, 88% at 5 years, and 78.6% at 10 years. During the follow-up period, there were two cardiac-related deaths in MV replacement group, 1 because of arrhythmia and 1 because of heart failure. The 1- and 5-year freedom from cardiac-related death rates were 85.7% and 64.3%, respectively, in the MV replacement group. In total, 3 patients died in the MV repair group, 1 because of arrhythmia and 2 because of heart failure. The 1-, 5- and 10-year freedom from cardiac-related death rates was 97.5%, 93.1%, and 82.7%, respectively, in the MV repair group (Fig. 2). A Kaplan–Meier survival analysis showed significant differences in the freedom from cardiac-related death rates (log rank *P* = 0.04).

**Fig. 3:**
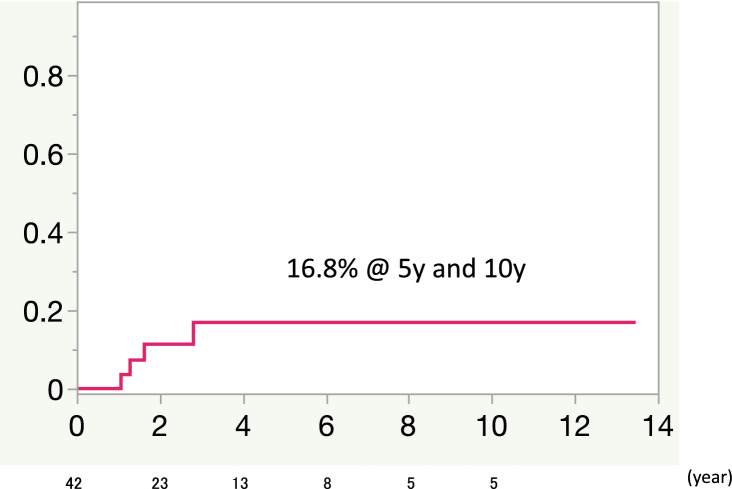
Recurrent mitral regurgitation (MR) during follow-up in the study group. The rate of recurrent MR was 16.8% at 5 and 10 years in the MV repair group. *MR* mitral regurgitation, *MV* *repair* mitral valve repair

**Fig. 4:**
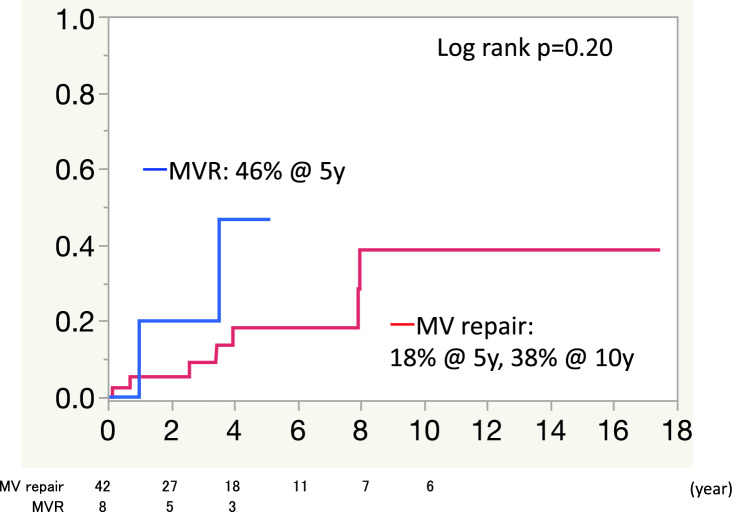
Recurrent heart failure rate in the study group. The recurrent heart failure rate was 46% at 5 years in the MVR group and 18% at 5 years and 38% at 10 years in the MV repair group with no significant differences (log rank *p* = 0.2). *MVR* mitral valve replacement, *MV repair* mitral valve repair

During the follow-up period, there was 1 patient who had moderate perivalvular leakage 3-year after MV replacement. The rate of recurrent MR was 16.8% at both 5 and 10 years in the MV repair group (Fig. 3). The mechanisms of recurrent MR were ring dehiscence after partial band annuloplasty in 1patient, and inadequate leaflet coaptation in 5 patients (partial band in 3 patients and full ring in 2 patients). There were 8 patients who suffered heart failure during the follow-up in MV repair group, 3 because of congestive heart failure, 1 because of mitral stenosis, 1 because of recurrent MR, 1 because of hypertension, and 1 because of bradycardia due to Afib. In MVR group, three patients suffered congestive heart failure during the follow-up. The recurrent heart failure rate was 46% at 5 years in the MV replacement group, 18% at 5 years, and 38% at 10 years in the MV repair group; the difference was not significant (Fig. 4) (*P* = 0.2). One patient in MV repair group suffered cerebral infarction 2 year after MV repair.

### Predictors of recurrent MR and heart failure in MV repair

Outcomes of univariate and multivariate analyses for the risk factors of recurrent MR in MV repair are presented in Table 4. Univariate analysis showed that the use of partial bands for mitral annuloplasty and the increase in preoperative LVESVI were risk factors for recurrent MR. Multivariate analysis showed that the only independent predictor for recurrent MR in the long term was the use of partial bands for mitral annuloplasty.

**Table 4:** Risk factor for recurrent MR and heart failure after mitral value repair

	Univariate analysis			Multivariate analysis		
	HR	95%CI	*P* value	HR	95%CI	*P* value
Risk factor for recurrent MR						
Partial band	17.7	1.8–171.2	0.01	10.1	1.00–103.2	0.049
LVESVI	1.09	1.00–1.24	0.03	1.07	0.96–1.26	0.19
MR grade 4	8.8	0.91–85.1	0.06	2.58	0.25–26.2	0.42
Risk factor for Heart failure						
LAVI	1.005	0.99–1.01	0.056			
Afib duration	1.07	0.98–1.16	0.1			
CKD	4.3	0.7–26.1	0.1			

*MR* mitral regurgitation, *LVESVI* left ventricular end-systolic volume index, *LAVI* left atrial volume index, *Afib* atrial fibrillation, *CKD* chronic kidney disease

In terms of the risk factor for heart failure in MV repair, univariate analysis showed that the increase in preoperative LA volume index, a longer history of AF, and the presence of chronic kidney diseases was associated with a higher risk of recurrent heart failure; however, this association was not statistically significant.

## Discussion

The current analysis of 50 patients with AFMR can be summarized as follows: (i) patients with severe preoperative MR, posterior leaflet tethering, and higher LVESVI tended to undergo MV replacement rather than MV repair; (ii) the use of partial bands for mitral annuloplasty was a strong predictor of recurrent MR after MV repair; and (iii) the freedom from cardiac death rate for all patients was 88% at 5 years and 78.6% at 10 years. Furthermore, the long-term freedom from cardiac death rate for MV replacement group was significantly lower than that of the MV repair group.

Balogh et al. [10] reported that the 5-year all-cause mortality and 5-year readmission rate for heart failure was 12% and 10%, respectively, after MV repair for AFMR; in addition, a previous study showed similar results [9]. The authors showed that the 5-year cardiac death rate and readmission rate after MV repair for heart failure was 6.9% and 18%, respectively, which appears to be an acceptable outcome when compared with the previous study.

In our study, the long-term freedom from cardiac death rate for the MV replacement group was significantly lower than that of the MV repair group. However, patients in the MV replacement group tended to experience severe preoperative MR, posterior leaflet tethering, and higher LVESVI, which indicated the MV replacement group showed advanced preoperative cardiac dysfunction compared to the MV repair group. Therefore, poorer outcomes for patients in the MV replacement group would be conceivable.

As mentioned above, the freedom from cardiac death rate for the MV repair group was higher than that of the MV replacement group in the long term. However, recurrent MR of a greater than mild-moderate degree has been reported in 20–35% of such patients 2–5 years after the MV repair [6, 9, 11], which suggest that MV repair is not sufficient for correcting MR in certain patients. Our study showed that the 5- and 10-year rates of recurrent MR were 16.8%. We identified that the use of partial bands for mitral annuloplasty was a strong predictor for recurrent MR after MV repair, which was consistent with the results of our previous study (12). Reportedly, a full ring impaired the natural annulus motion of the MV and increased the gradient across the valve (13–15). However, using a full ring could help maintain the mitral annulus in its original form and increase the length of leaflet coaptation compared to partial band, thereby preventing recurrent MR (12).

Although the univariate analysis identified high LVESVI and preoperative severe MR as risk factors for recurrent MR after MV repair, these factors were not identified as independent predictors for recurrent MR in the multivariate analysis. However, a high LVESVI and severe MR are still important factors. Sakaguchi et al. (6) reported significantly larger preoperative LVDd and LVDs in patients with recurrent MR after mitral annuloplasty for AFMR during follow-up.

AFMR is the result of an enlarged left atrium and insufficient leaflet remodeling with preserved LV function. However, in actual clinical settings, patients with atrial MR can also experience mild LV dilatation or mild reduced systolic function owing to volume overload resulting from chronic MR, especially in the advanced stages (16). Other studies have reported the presence of a relationship between LV dilatation and worsening atrial MR (17–19). Progressive LA remodeling and LV dilatation with MR-induced volume overload cause progressive mitral annular dilatation and leaflet tethering in advanced stages, which can deteriorate AFMR (2, 20). The authors concluded that mitral annuloplasty alone might be insufficient for achieving long-term correction of MR in patients with combined AFMR, left ventricular dilatation, and excessive leaflet tethering in advanced stages (2, 6). In our study, higher LVESVI and severe MR were relatively higher risk factors for recurrent MR after MV repair, which also indicated that MV repair might not be appropriate for advanced AFMR and that early intervention for AFMR before reaching advanced stages would be required to prevent recurrent MR and cardiovascular events in the long term.

## Limitations

Our study has several limitations. First, the study was retrospective and observational and included a relatively small number of patients. Second, the surgical procedures were selected by an individual surgeon as the optimal procedure at the time; therefore, there were variations in prosthesis selection. Reportedly, posterior leaflet extension is effective for AFMR (5); however, this procedure was not performed for AFMR in this study. Third, there were significant differences in preoperative cardiac function between the patients undergoing MV repair and those undergoing MV replacement. Ideally, we should not compare between such 2 groups because the mid-term outcomes could have been influenced by a bias related to patient background. However, we would like to indicate that MVR could not improve outcome in patients with AFMR if MR and LV dilatation progressed. Further studies that minimize the patient bias are warranted. Fourth, to assess the AFMR in detail, we employed tenting height, tethering angle, and leaflet area. However, owing to the considerable lack of data on these parameters, we could not include important parameters in this analysis. More detailed analysis that includes these parameters would be required to identify the mechanism of recurrent MR. Fifth, as some previous study of AFMR include patients with paroxysmal Afib (17, 21–23), our study includes patients with paroxysmal Afib preoperatively. Potentially, patients background would be different between patients with persistent Afib and paroxysmal Afib, which might influence patient outcome. However, we investigated backgrounds, surgical outcome, cardiac-related death, and rate of recurrent heart failure in the AFMR cohort excluded patients with paroxysmal Afib (Supplemental Table, Supplemental Fig. 1, 2, 3), which showed similar results to this study.

## Conclusion

Patients who underwent MV repair for AFMR could have an acceptable mid-term outcome. MVR was indicated for the patients who had higher LVESVI and severer MR grade than patients who underwent MV repair. However, MVR could not improve the mid-term outcome in those patients. In terms of recurrent MR mid-term after MV repair, the use of partial bands for mitral annuloplasty would not be recommended.

## Supplementary Information

Below is the link to the electronic supplementary material.

Supplementary file1 (PDF 78 KB)

Supplementary Fig. 1. Freedom from cardiac death in the study group excluding patients with paroxysmal Afib. The 1- and 5-year freedom from cardiac-related death rates was 80% and 60%, respectively, in the MVR group. The 1-, 5-, and 10-year freedom from cardiac-related death rates was 96%, 91.5%, and 78.5%, respectively, in the MV repair group (log rank p = 0.04). Afib: atrial fibrillation, MVR: mitral valve replacement, MV repair: mitral valve repair (PDF 49 KB)

Supplementary Fig. 2. Recurrent mitral regurgitation (MR) during follow-up in the study group excluding patients with paroxysmal Afib. The rate of recurrent MR was 16.3% at 5 and 10 years in the MV repair group. Afib: atrial fibrillation, MR: mitral regurgitation, MV repair: mitral valve repair (PDF 37 KB)

Supplementary Fig. 3. Recurrent heart failure rate in the study group excluding patients with paroxysmal Afib. The recurrent heart failure rate was 50% at 5 years in the MVR group and 22% at 5 years and 48% at 10 years in the MV repair group with no significant differences (log rank p = 0.23). Afib: atrial fibrillation, MVR: mitral valve replacement, MV repair: mitral valve repair (PDF 40 KB)
