# Supplementary material for: Mitral valve surgery for atrial functional mitral regurgitation: predicting recurrent mitral regurgitation and mid-term outcome
Source: Gen Thorac Cardiovasc Surg. 2022 Mar 3;70(9):761–9. doi: 10.1007/s11748-022-01793-8 (PMC9371988; doi:10.1007/s11748-022-01793-8)
Supplement: Supplementary file 1 — Supplementary file1 (PDF 78 KB) [file 11748_2022_1793_MOESM1_ESM.pdf]

Supplemental Table. Backgrounds and postoperative findings in the study group excluding patients with paroxysmal Afib

	All	MV repair	MVR	P value
Number of patients	42	35	7	
Age	71 (68.5-77.5)	70 (66.5-75)	73 (67.5-78.5)	0.78
Male	25 (59.5%)	22 (62.8%)	3 (42.8%)	0.55
MR grade 4	15 (%)	10 (24%)	5 (71%)	0.06
LVDd (mm)	58±9.4	57±7.4	62±8.2	0.21
LVEDVI (ml/m^2)	110±39	105±37	133±37	0.07
LVDs (mm)	39±7.7	37±6.8	43±8.7	0.17
LVESVI (ml/m^2)	45±20	39±16	57±21	0.04
LA dimension (mm)	65 (57-73)	65 (55-74)	63 (56-73)	0.92
TRPG (mmHg)	34±12	33±12	32±13	0.8
EF (%)	67±11	70±7.8	67±7.7	0.21
TR≥3	17 (40%)	13 (31%)	4 (57%)	0.17
Mitral annular dilation	42 (100%)	35 (100%)	7 (100%)	1
Posterior leaflet tethering	11 (26%)	7 (20%)	4 (57%)	0.03
Postoperative echocardiographic findings				
MR≥2	0	0	0	
LVDd (mm)	54 (49-60)	52±7.2	60±8.4	0.21
LVDs (mm)	39 (34-46)	38±7.4	48± 8.0	0.025
LA dimension (mm)	54 (44-61)	54±11	58±14	0.77
TRPG (mmHg)	23 (19-26)	23±8.1	24±4.2	0.93
EF (%)	50 (40-60)	51±11	43±16	0.57
Postoperative course				
Hospital death	1 (2.4%)	0	1 (14.2%)	0.04
Respiratory failure	2 (4.8%)	1 (2.8%)	1 (14.2%)	0.59
Cerebrovascular event	1 (2.4%)	1 (2.8%)	0	0.59
Reexploration	2 (4.8%)	1 (2.8%)	0	0.59
Renal failure	1 (2.4%)	1 (2.8%)	0	0.59
