# Supplementary figures and images for: Mitral valve surgery for atrial functional mitral regurgitation: predicting recurrent mitral regurgitation and mid-term outcome
Source: Gen Thorac Cardiovasc Surg. 2022 Mar 3;70(9):761–9. doi: 10.1007/s11748-022-01793-8 (PMC9371988; doi:10.1007/s11748-022-01793-8)

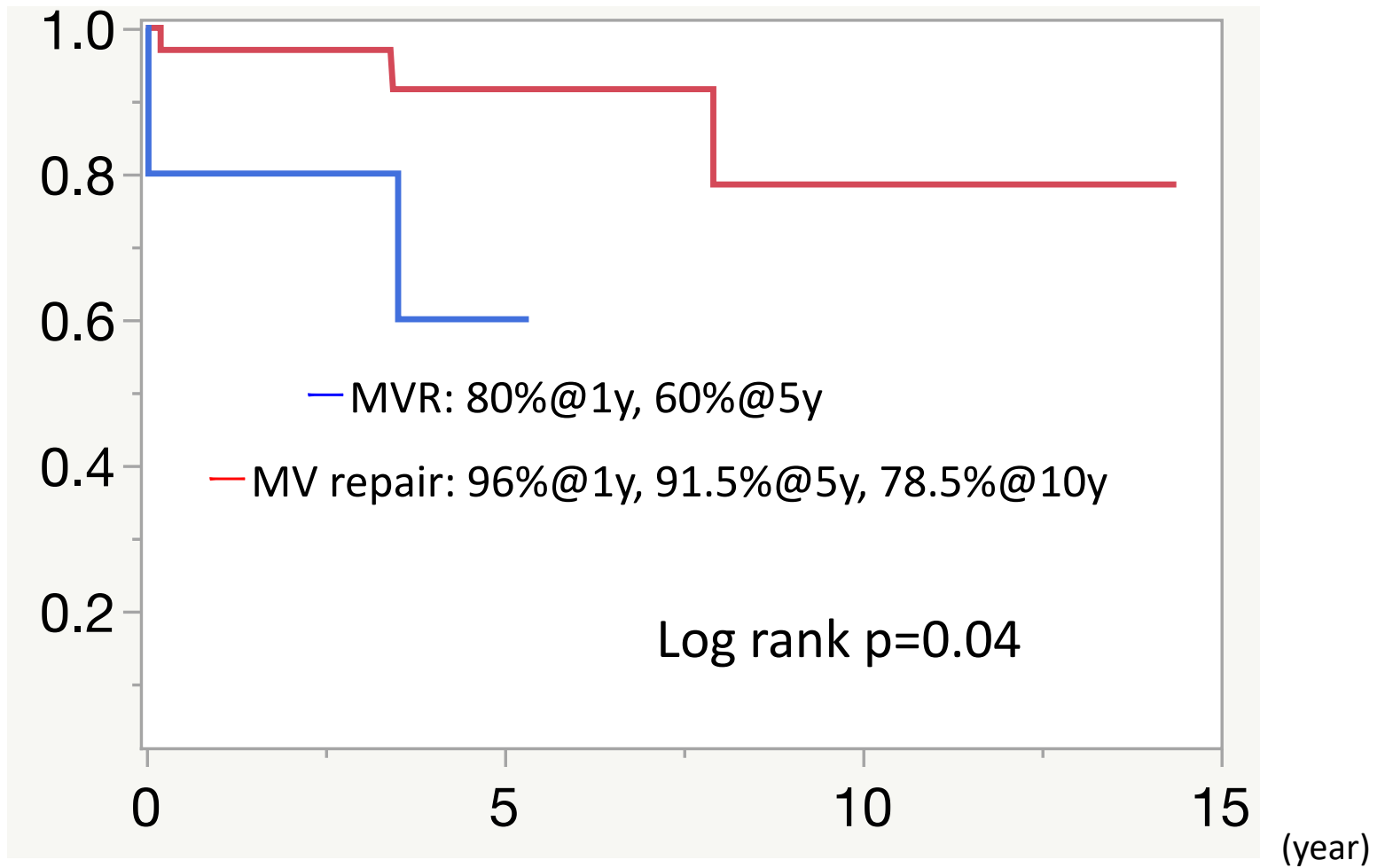

|           |    |    |    |   |
|-----------|----|----|----|---|
| MV repair | 35 | 21 | 12 | 6 |
| MVR       | 7  | 4  | 2  |   |

Supplement: Supplementary file 2 — Supplementary Fig. 1. Freedom from cardiac death in the study group excluding patients with paroxysmal Afib. The 1- and 5-year freedom from cardiac-related death rates was 80% and 60%, respectively, in the MVR group. The 1-, 5-, and 10-year freedom from cardiac-related death rates was 96%, 91.5%, and 78.5%, respectively, in the MV repair group (log rank p = 0.04). Afib: atrial fibrillation, MVR: mitral valve replacement, MV repair: mitral valve repair (PDF 49 KB) [file 11748_2022_1793_MOESM2_ESM.pdf]

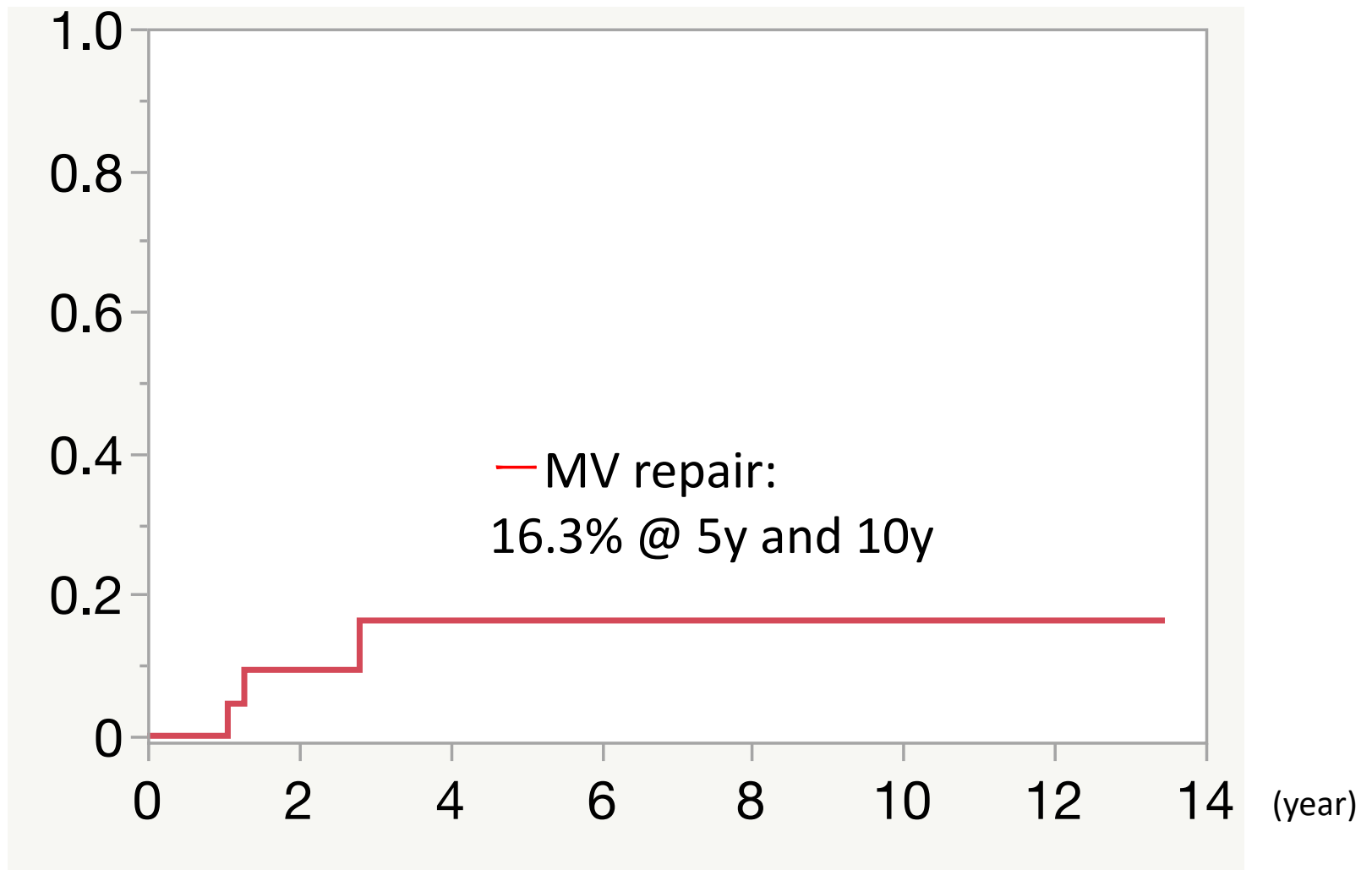

MV repair

35

11

8

4

4

Supplement: Supplementary file 3 — Supplementary Fig. 2. Recurrent mitral regurgitation (MR) during follow-up in the study group excluding patients with paroxysmal Afib. The rate of recurrent MR was 16.3% at 5 and 10 years in the MV repair group. Afib: atrial fibrillation, MR: mitral regurgitation, MV repair: mitral valve repair (PDF 37 KB) [file 11748_2022_1793_MOESM3_ESM.pdf]

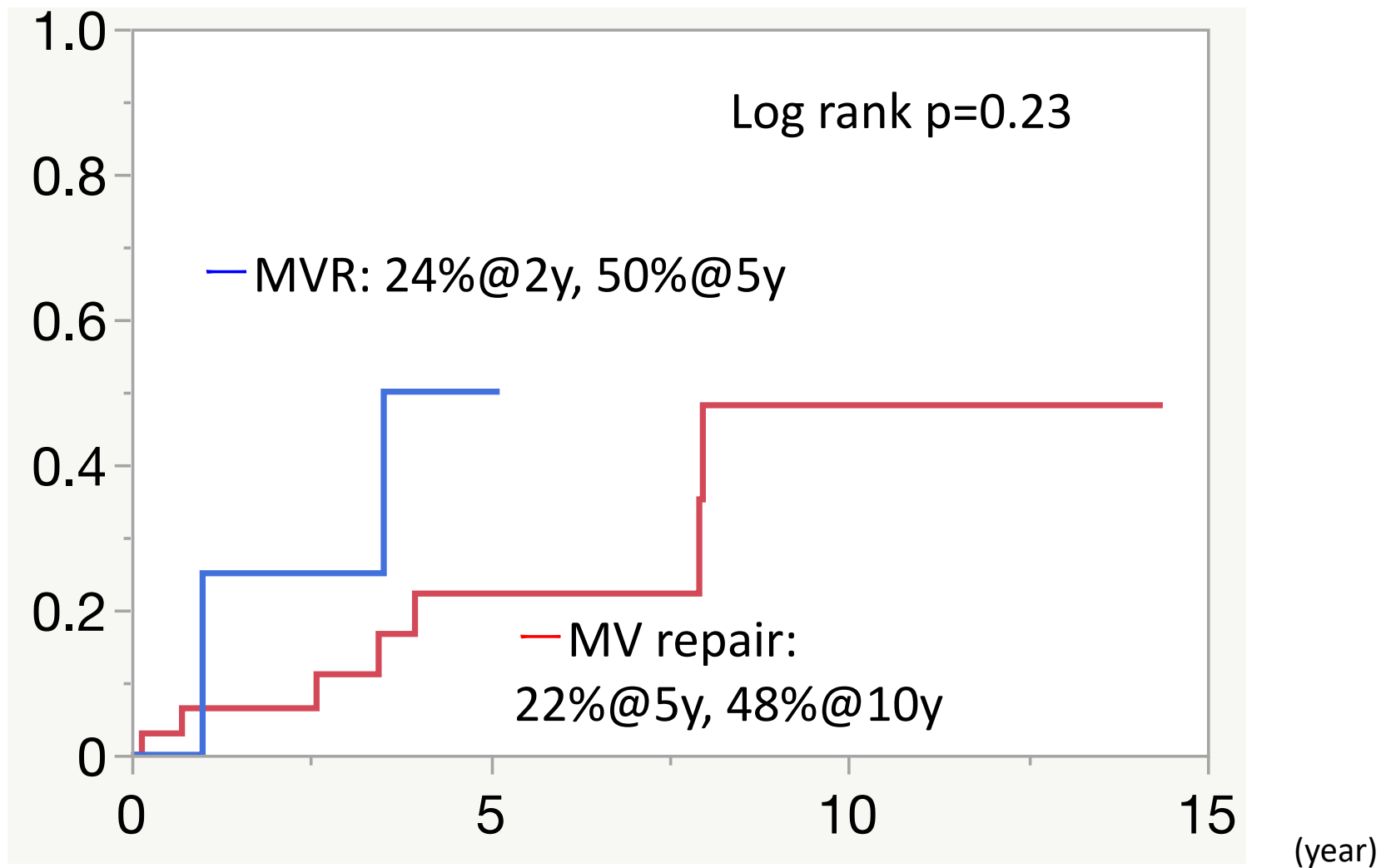

MV repair  
MVR

35

19

9

74

7

4

2

Supplement: Supplementary file 4 — Supplementary Fig. 3. Recurrent heart failure rate in the study group excluding patients with paroxysmal Afib. The recurrent heart failure rate was 50% at 5 years in the MVR group and 22% at 5 years and 48% at 10 years in the MV repair group with no significant differences (log rank p = 0.23). Afib: atrial fibrillation, MVR: mitral valve replacement, MV repair: mitral valve repair (PDF 40 KB) [file 11748_2022_1793_MOESM4_ESM.pdf]
